# Computer-Assisted Protocol-Adherent Blood Lipid Evaluation in Vascular Outpatients (CAPABLE-Vascular)

**DOI:** 10.3390/jcm14041321

**Published:** 2025-02-17

**Authors:** Benjamin Thurston, Shrirajh Satheakeerthy, Lewis Hains, Andrew E. C. Booth, Christina Gao, Jamie Bellinge, Brandon Stretton, Peter Psaltis, Stephen Bacchi

**Affiliations:** 1 Faculty of Health and Medical Sciences, The University of Adelaide, Adelaide, SA 5005, Australia; benjamin.thurston@sa.gov.au (B.T.); shrirajh.satheakeerthy@sa.gov.au (S.S.); lewis.hains@student.adelaide.edu.au (L.H.); andrew.booth@sa.gov.au (A.E.C.B.); christina.gao@student.adelaide.edu.au (C.G.); brandon.stretton@sa.gov.au (B.S.); peter.psaltis@sa.gov.au (P.P.); 2 Department of Nuclear Medicine, School of Medicine, Sir Charles Gairdner Hospital, University of Western Australia, Crawley, WA 6009, Australia; jamie.bellinge@health.wa.gov.au; 3 Neurology Department, Lyell McEwin Hospital, Haydown Road, Elizabeth Vale, SA 5112, Australia; 4 College of Medicine and Public Health, Flinders University, Bedford Park, SA 5042, Australia

**Keywords:** digital health, automation, efficiency, stroke, cardiovascular

## Abstract

**Background:** The lack of availability of test results in vascular surgery outpatient clinics impedes the medical management of vascular risk factors, such as dyslipidaemia and diabetes mellitus. This study sought to evaluate the feasibility of using computer-assisted processes to promote the ordering of routine investigations to promote this management. **Method:** After consultation with specialist clinicians, clinician–programmers developed a rule-based system to facilitate the ordering of lipid studies and HbA1c prior to vascular clinic appointments. A four-week historical control period prior to the initiation of the intervention was compared to a four-week period following the intervention. **Results:** There were 1165 patients in the study. In the pre-intervention period, 38.0% of patients had HbA1c and 17.9% had lipid studies in the preceding 6 months. In the post-intervention period, HbA1c and lipid studies were ordered for 100% of vascular outpatients (*p* < 0.001). **Conclusions:** The use of computer-assisted processes to facilitate the requesting of routine outpatient investigations is feasible and shows early signs of being effective. Follow-up studies examining clinical endpoints are required.

## 1. Introduction

Treatment of dyslipidaemia and diabetes mellitus, in particular type 2 diabetes mellitus (T2DM), is integral to the management of vascular disease [1]. The vascular surgeon can therefore play an important role in medical management. However, with busy clinics, risk factor modification may not be adequately addressed—especially when the necessary blood tests are not performed prior to the appointment [2]. Automation could improve the efficiency and completeness of these services.

Despite advancements in healthcare delivery, the increasing burden of chronic diseases like T2DM and dyslipidaemia continues to challenge the capacity of outpatient clinics. Approximately 1.3 million Australians and 537 million people globally live with T2DM, with the prevalence of microvascular complications such as diabetic nephropathy and retinopathy affecting approximately 25% of these patients [3,4]. The prevalence of macrovascular complications such as peripheral vascular disease and myocardial infarction is estimated to be between 6 and 10% [5]. The prevalence of dyslipidaemia is significantly higher in Australia, affecting 63% of Australians [6]. This issue is highlighted in vascular surgery clinics. The management of these major contributors to vascular disease is a necessary component of the care of patients with vascular pathology. The increasing complexity of healthcare systems, combined with growing patient loads, underscores the need for innovative solutions to streamline diagnostic and management workflows. Addressing this gap is critical to ensuring that high-risk patients receive timely and effective care.

Globally, there is an increasing burden of T2DM and dyslipidaemia, both of which contribute to the increasing burden of subsequent micro- and macrovascular complications. With over 12% of the population estimated to live with T2DM by 2045, clinicians will have an increasing responsibility to manage T2DM and its complications [7]. The pathogenesis of micro- and macrovascular complications of T2DM are largely multifactorial, with various contributing mechanisms that underlie these disease processes. Almost all of these mechanisms are driven by the hyperglycaemia and insulin resistance seen in T2DM [8]. Macrovascular complications such as stroke and myocardial infarction result largely from increased atherosclerotic plaque accumulation as a consequence of a systemic pro-inflammatory state and endothelial dysfunction from chronic hyperglycaemia [8]. Similarly, microvascular complications such as diabetic nephropathy and retinopathy result from chronic low-grade inflammatory response from hyperglycaemia-induced increases in advanced glycation end products, oxidative stress, reactive oxygen species, and hypoxia [9].

While patients with vascular disease often have multiple medical specialists due to the complexity of their cardio-metabolic syndrome, some patients only have contact with vascular surgeons and for many patients the diagnosis of peripheral arterial disease alters their risk profile and therefore optimal preventative strategies. Furthermore, the nature of HbA1c, blood sugar levels (BSLs), and lipid testing typically precludes on-the-spot diagnosis and management of T2DM and dyslipidaemia. The standard clinical pathway for testing, requiring an initial appointment with subsequent appointments to review results, contributes to this delay. With increasing outpatient wait times locally, this may further contribute to delayed risk factor management.

Multidisciplinary care is a necessity in the management of patients with significant vascular pathology. Namely, patients with vascular disease often require input from multiple specialists, including endocrinologists, cardiologists, and vascular surgeons, to address their complex cardio-metabolic profiles. Yet, in many settings, the vascular surgeon may be the only consistent point of contact for these patients, particularly in the context of peripheral arterial disease. In low-resource settings, or for patients with limited engagement with healthcare, this single point of contact being the vascular surgeon may be more common. This potential places an additional burden on surgeons to address systemic risk factors, a task that could be optimised through the integration of automated systems to pre-emptively identify gaps in care, such as overdue blood tests for lipid and glycaemic control.

Modern technological approaches, including automation, may be able to help address this issue. Automation is widely proposed and utilised to help improve areas such as optimising healthcare workflow processes and systems. These technologies have a particularly useful role in chronic disease management in alleviating administrative burdens and decreasing clinical workload, without removing human clinical oversight [10].

Technological advancements, particularly in automation and artificial intelligence, hold significant potential to transform routine clinical workflows. However, futuristic artificial intelligence algorithms are not necessarily required to improve existing workflows. Simple technological fixes can improve a number of outcomes when deployed strategically [11,12,13]. However, their application to chronic disease management remains underutilised. By leveraging automated systems to identify patients with overdue investigations and streamline the ordering process, clinics could mitigate delays in care, improve patient outcomes, and enhance clinic efficiency. This study seeks to build on this potential by exploring the feasibility of developing a computer-assisted process to improve pre-appointment evaluations, with a focus on T2DM and dyslipidaemia, in a vascular outpatient setting.

The aims of this study were to examine the feasibility of developing a computer-assisted process to facilitate the evaluation of dyslipidaemia and T2DM in vascular outpatients. Computer-assisted technologies and workflow algorithms such as this one have the potential for widespread deployment and implementation across various outpatient specialties and potentially primary care centres to automate simple clinical workflows.

## 2. Materials and Methods

**Figure 1:**
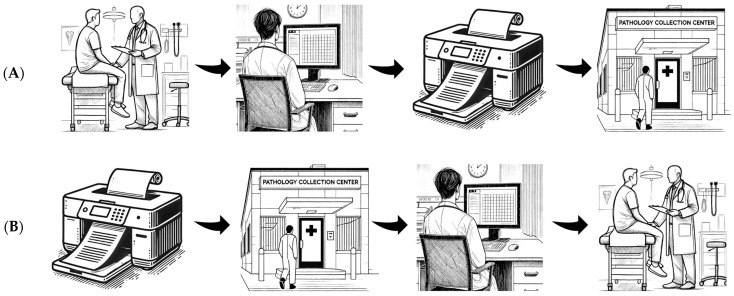
Routine practice (**A**, **top**) and intervention (**B**, **bottom**) workflows.

This study was conducted in the outpatient departments of the Northern and Central Adelaide Local Health Networks, which encompass four hospitals. Routine practice (Figure 1A) involves surgeons in outpatients reviewing lipid studies and HbA1c during a patient’s appointment and requesting further blood tests if not available. Often, vascular surgeons suggest the patient reviews the blood tests with their general practitioner, particularly if further vascular surgery review is not warranted, and this shifts risk factor modification from the specialist to the generalist.

The study intervention (Figure 1B) involved the development of a computerised process to facilitate pre-appointment assessment of dyslipidaemia and T2DM. Initially, clinician–programmers discussed the problems faced by vascular and cardiology specialists. These multidisciplinary discussions included a review of existing workflows and shortcomings in service provision. Through the review of these real-world challenges with vascular and cardiology specialists, a sound grasp of the problem at hand and a shared model of possible solutions were able to be reached. The possible solutions that were discussed in this multidisciplinary planning phase directly informed the developed intervention.

An automated rule-based system was then developed. This system ascertains which patients will be seen in vascular outpatients between one and five weeks from the date that the program is run. This process is completed through automated review of upcoming clinic bookings. The program then determines whether these individuals have had lipid studies and HbA1c checked within six months. This determination is made on the basis of automated analysis of each patient’s pre-existing pathology records. If patients have not had these tests checked, a pathology form is autocompleted for these tests. A clinician reviews and signs these before being sent to patients, prior to their appointment.

This study employed a pre- and post-implementation design. All adult patients with appointments in the selected vascular surgery clinics during the study period were included. These patients were selected not due to a history of T2DM and dyslipidaemia, but rather because it is routine protocolised practice for all patients seen in these clinics to have these tests performed. Four weeks prior to the initiation of the intervention served as a control period. This period was compared to the four weeks following the intervention. The primary outcome was the number of requested HbA1c and lipid study tests.

We assessed a sub-cohort of 100 patients for potential blood test duplication rates. For these patients, we manually searched all providers in South Australia and contacted the patients’ GPs to obtain results. The focus was on duplication of blood tests sent within the last 6 months.

Descriptive statistics were used to present cohort characteristics. Data were inspected for normality. Chi-squared tests, Fisher exact tests, Mann–Whitney-U tests, and unpaired *t*-tests were used as appropriate to compare between the pre- and post-implementation groups. For the primary results of interest, comparing the proportion of patients in the pre-intervention period and the post-intervention period who had a request for HbA1c and lipid studies, chi-square and Fisher exact tests were used. The statistical significance level was set at *p* < 0.05. Analysis was conducted in Python and R.

This study was conducted with ethics approval from the Human Research Ethics Committee of the Central Adelaide Local Health Network, with a waiver of individual consent.

## 3. Results

There were 1165 patients in this study. The mean age was 68.4 years (standard deviation 15.0). The number of female patients in this study was 418/1165 (35.9%).

In the pre-intervention period, the number of patients with HbA1c requested was 246/647 (38.0%) and lipid studies was 116/647 (17.9%). Following the initiation of the intervention, the number of outpatients with HbA1c and lipid studies requested were 518/518 (100%, *p* < 0.001) and 518/518 (100%, *p* < 0.001), respectively.

Of the 100 patients assessed for duplication of tests, 34 had an HbA1c and 21 patients had a lipid profile at SA Pathology, with 12 and 8 patients, respectively, having had these tested in other providers. Of these, five patients were common to both groups, so in total 15% of patients would have had at least one test duplicated following intervention.

## 4. Discussion

Through a relatively simple programmatic intervention, adherence to the ordering of tests for vascular risk factors was improved. While this work is still in a pilot or preliminary stage, these results demonstrate that such an approach is feasible. This type of approach may have value in other specialty domains such as endocrinology and potentially general practice. The application of this technology to other routine testing and specialty areas may further broaden its potential for wider implementation.

Testing metabolic health markers prior to outpatient appointments will likely vastly increase outpatient care efficiency and improve the management of vascular comorbidities associated with dyslipidaemia and T2DM. Anticipating routine pathology testing prior to outpatient appointments may also contribute to significant healthcare cost savings, both in reducing the number of outpatient appointments required and through swift management of dyslipidaemia and T2DM to avoid vascular complications. Whilst this tool would initially be associated with increased healthcare costs, cost savings in reducing complications such as stroke and other vascular conditions would potentially result in vast long-term savings.

Developing such automation requires in-depth knowledge of clinical workflows. This knowledge is not just at a system level, but requires local expertise, as workflows may vary significantly between specialties and locations. This need for workflow knowledge highlights the value of clinician–programmers—health professionals who combine clinical expertise with programming skills. These individuals are uniquely positioned to identify inefficiencies in care pathways, design tailored automation solutions, and ensure effective integration with existing workflows. Cultivating this hybrid skillset among clinicians will be critical to advancing the adoption of automation in healthcare and ensuring these tools are both effective and user-friendly. Supporting individuals with this skillset will also clearly be integral to the adoption of artificial intelligence in healthcare.

In order to cultivate clinician–programmers, accredited time during specialty training must be provided to enable the development of programming and digital health skills [14]. Specialty training programs are time-intensive. However, it is now necessary to realise that this skillset is a requirement for some practitioners, and that it is necessary to create opportunities for the development of skills in the realm of digital health for certain trainees. Dedicated or protected time within training curricula for skill development could bridge this gap, fostering a new generation of clinician–programmers who can lead innovation in clinical workflow automation. In the long term, this investment would yield significant dividends by improving healthcare efficiency and outcomes.

There were several pragmatic considerations that fed into the design of this study and the evaluation of the results, in particular with respect to outside pathology providers. In the participating hospitals, SA Pathology laboratory tests are available through the electronic medical record. Pathology tests from outside providers may at times be visible in My Health Record; however, in the clinical setting, the loading times and usability of this viewer precludes its use without compromising other areas of patient care. Accordingly, if results were not present from SA Pathology, they were considered to be absent, highlighting the importance of user experience and workflow integration for electronic medical records [15]. This approach could result in duplicate blood tests, which are best avoided, but our sub-cohort analysis suggested a duplication rate of less than 15%, which was deemed clinically acceptable [16,17]. Furthermore, integration of these algorithms into EMR systems comes with its own challenges, with many system restrictions impeding their direct implementation into the EMR. Whilst challenging, direct implementation into the EMR may help reduce test duplication rates and further increase the cost-effectiveness of this technology. Further reductions in test duplication may be accomplished through system integration into shared health systems, accessible by both primary care and specialist physicians, such as Australia’s My Health Record.

Further improvement of this technology may require integration of local clinical practice guidelines to prompt pathology ordering. For example, the integration of the American College of Cardiology lipid management guidelines may autogenerate pathology requests at 4–12 weeks post-commencing statin therapy, with repeated testing at 3–12 months, hence increasing efficiency and reducing the likelihood of missed pathology testing [18]. Similarly, this can be integrated into HbA1c testing, which current local guidelines recommend be tested every 6–12 months depending on the clinical situation [19,20]. This is particularly important in managing T2DM, as the current literature has reported strong associations between HbA1c testing guideline adherence and improved glucose control and lower incidence of complications such as Chronic Kidney Disease [19]. For optimal integration, this would require dual integration between specialist and general practice clinics to guide best management.

Our study has used the request for a pathology test as an indicator of process [21]. However, not all pathology tests that are requested are performed, and not every test result that is returned is treated appropriately. Similar criteria-based workflows and physician education solutions have been explored in the cardiology outpatient setting to avoid unnecessary investigations that could similarly be integrated into EMR workflows [22,23,24,25,26,27]. Ongoing research should examine the impact of the developed system, particularly the number of patients who obtain a timely result from the pathology request. Surrogate endpoints could be evaluated, including follow-up lipids and HbA1c. However, the best measure of the program’s effectiveness would be major clinical endpoints, such as rates of stroke, myocardial infarction, and death. These outcomes would also serve as the best measure of the tool’s cost effectiveness. Evaluation in the postoperative period [28] and in other specialties may also be beneficial. Evaluation of patient-reported measures evaluating health system satisfaction may also reveal the further benefits of this technology through increasing outpatient efficiency and improving healthcare outcomes.

Studies evaluating the implementation of an intervention, such as this one, do not always require randomization. Randomised controlled trials (RCTs) are the gold standard for evaluating interventions that involve potential confounding variables or where the outcomes are subject to significant bias. However, in this study, randomisation was not necessary, as the primary goal was to assess the feasibility of an automated process. Additionally, there were no substantial risks with the intervention, and minimal cost relative to the ideal standard of care that would already be provided. Further integration may also seek to provide guideline-based suggestions on necessary medication modification through guideline decision-making workflows. The pre- and post-implementation design allowed for a straightforward evaluation of the intervention’s impact on test ordering rates without introducing unnecessary complexity.

Successful implementation of automation in healthcare requires careful consideration of the various stakeholders and their differing needs and goals. Poorly automated workflows can negatively impact quality and trust [29,30]. Notably, the doctor–patient relationship serves a foundational role in healthcare delivery [31], and thus workflows selected for automation must maintain trust in the professionals and technology involved. The degree of automation should be carefully considered, based upon the need for human input or review at critical workflow decision points [32]. Additionally, ensuring patient confidentiality and data security are crucial considerations in healthcare [33], necessitating careful planning and protection for highly automated workflows. Finally, when utilizing and implementing advanced computational techniques in healthcare, additional care must be taken so as to not perpetuate or create further biases [34].

The limitations of this study include that all centres were within a single state. The generalisability of the approach will require evaluation elsewhere. Although the details of the programming may differ, the underlying workflow could be essentially unchanged.

Future automation of additional simple clinical workflows may be highly effective in the optimisation of healthcare systems. Automation of certain healthcare tasks such as operational pharmacological processes has already shown to be both effective and efficient [30]. Additionally, initial investigations assessing automation of clinical care tasks such as application of clinical guidelines [35] and transmitting results [36] have been favourably perceived. Particularly, the implementation of workflow algorithms can be leveraged to help alleviate administrative workload, ultimately enhancing healthcare efficiency and improving patient outcomes [37]. In addition, the automation of clinical data review could be implemented alongside the suggested processes, to identify gaps and locate data, or resolve duplicates from multiple sources [32]. This could further support reducing the burden of cognitive tasks required for successful treatment and care delivery workflows.

## 5. Conclusions

The development of computer-assisted processes to promote the monitoring of routine vascular risk-factors is feasible and shows early signs of effectiveness. Further studies examining relevant clinical endpoints such as stroke and myocardial infarction are required. This approach may have value for other outpatient departments that require routine investigations prior to clinic appointments such as endocrinology as well as utilisation in the primary care setting.

## Data Availability

The original contributions presented in this study are included in the article. Further inquiries can be directed to the corresponding author.
